# *Clostridioides difficile* toxin is infrequently detected in inflammatory bowel disease and does not associate with clinical outcomes

**DOI:** 10.1186/s13099-022-00511-2

**Published:** 2022-08-30

**Authors:** Rachel Bernard, Muhammad B. Hammami, Forest W. Arnold, Brian Mcgrath, Alieysa Patel, Brandon Wuerth, Maribeth R. Nicholson, Krishna Rao, Dejan Micic

**Affiliations:** 1 Division of Pediatric Gastroenterology, Hepatology and Nutrition, Department of Pediatrics, Monroe Carell Jr. Vanderbilt Children’s Hospital, Nashville, TN USA; 2 Division of Gastroenterology and Hepatology, Department of Medicine, VA Loma Linda Healthcare System, Loma Linda, CA USA; 3 Department of Medicine, University of California Riverside, Riverside, CA USA; 4 Division of Infectious Diseases, Department of Medicine, University of Louisville, Louisville, KY USA; 5 Division of Gastroenterology, Hepatology and Nutrition, Department of Medicine, University of Louisville, Louisville, KY USA; 6 Division of Infectious Diseases, Department of Medicine, University of Michigan Medical School, Ann Arbor, MI USA; 7 Section of Gastroenterology, Hepatology and Nutrition, Department of Medicine, University of Chicago Medicine, 5841 S. Maryland Ave. MC4076, Chicago, IL 60654 USA

**Keywords:** Inflammatory bowel disease, *Clostridium difficile*, Colitis

## Abstract

**Introduction:**

*Clostridioides difficile* infection (CDI) rates and outcomes can vary based on differences in testing strategy. Our aim was to assess the prevalence of toxin detection in inflammatory bowel disease (IBD) when compared to those without IBD. Secondly, the clinical outcomes of CDI in IBD were assessed using two-step testing strategies.

**Methods:**

We included patients undergoing CDI testing from four academic centers in the United States between January 1, 2018 and June 30, 2020. First the prevalence of toxin detection was compared between individuals with IBD and those without IBD. Secondly, among patients with IBD a primary composite outcome of abdominal colectomy, admission to an intensive care unit (ICU) or death within 30 days of *C. difficile* testing was assessed across the three categorical groups (screen positive/toxin positive, screen positive/toxin negative and screen negative assay) resulting from the two-step testing strategy.

**Results:**

When comparing individuals with a positive screening assay, patients with IBD were less likely to have toxin detected by enzyme immunoassay (EIA) as compared to the non-IBD population (22/145 (15.2%) vs. 413/1144 (36.1%), p < 0.0001). Among all patients with IBD (n = 300), twenty-five (8.3%) had a screen positive assay/toxin positive assay, 136 (45.3%) had a screen positive/toxin negative assay and 139 (46.3%) had a negative screening assay. No significant difference in the primary composite outcome was detected across the three groups (p = 0.566).

**Conclusion:**

When compared to those without IBD, patients with IBD have a reduced proportion of cases of *C. difficile* with toxin positivity. Differences in clinical outcomes among patients with IBD were not detected and limited by the infrequent detection of expressed toxin.

**Supplementary Information:**

The online version contains supplementary material available at 10.1186/s13099-022-00511-2.

## Introduction

*Clostridioides difficile* is a gram positive spore-forming bacteria which has become an increasingly recognized enteric pathogen affecting patients with inflammatory bowel disease (IBD) [1]. Asymptomatic carriage of *C. difficile* has been reported at an increased frequency in IBD with a diversity pattern reflecting community acquisition [1].

Between 1998 and 2004, rates of *C. difficile* infection (CDI) doubled in individuals with Crohn’s disease (CD) and tripled in those with ulcerative colitis (UC) [2]. Congruent with changes in CDI incidence have been changes in testing practices away from the detection of expressed *C. difficile* toxins to nucleic-acid amplification tests (NAAT) detecting the presence of toxigenic *C. difficile*. As NAAT are associated with increased sensitivity and potentially overdiagnosis [3], two-step testing with a screening assay (glutamate dehydrogenase (GDH) or NAAT) followed by a toxin detection test by enzyme immunoassay (EIA) are now increasingly recommended over a NAAT assay alone for CDI diagnosis [4]. Our aim was therefore to first assess for differences in the prevalence of toxin detection between screening strategy positive patients with IBD and control patients without IBD and then to evaluate for differences in clinical outcomes among individuals with IBD diagnosed using direct toxin detection when two-step testing is clinically performed.

## Methods

### Study design and assays

This was a multicenter, retrospective cohort study between the dates January 1, 2018 to June 30, 2020. The electronic medical records (EMR) were queried for positive cases of *C. difficile* among adult patients with a diagnosis of IBD (ICD-10: CD K50.x or UC K51.x) from four academic centers in the United States. Retrospective chart review was performed to confirm IBD diagnosis, obtain laboratory values within 48 h of CDI testing, and identify hospitalizations and surgeries within 30 days of CDI. Control cases without IBD seeking medical care for diarrhea were available from three participating centers.

### *C. difficile* infection testing

All centers used a two-step testing algorithm in which an initial screening assay for either glutamate dehydrogenase (GDH) or detection of target toxin genes by NAAT was used. The second step of the testing algorithm was performed to confirm the presence of toxin in the stools of positively screened patients by enzyme immunoassay (EIA). Therefore, patients could be separated into the categories: screen positive/toxin positive, screen positive/toxin negative or negative screening assay. Details of the two-step testing assays and cohort creation are described in the Additional Methods (see Additional file 1).

### Primary and secondary outcomes

This study consisted of two separate analyses. First the prevalence of toxin detection among positively screened patients was compared between individuals with IBD and the general population control patients without IBD. Secondly, individuals with IBD were assessed for outcomes related to *C. difficile* toxin detection. The primary composite outcome of interest in patients with IBD included abdominal colectomy, admission to an intensive care unit (ICU) or death (from any cause) within 30 days of *C. difficile* testing among patients with IBD. Secondary outcomes of interest included the need for hospitalization or readmission, differences in laboratory values and CDI management (choice of antibiotic therapy). Laboratory values assessed included: white blood cell count (WBC), hemoglobin, albumin and C-reactive protein (CRP) within 48 h of CDI testing. Escalation of immunosuppressive therapy was considered if initiation of systemic steroids ≥ 20 mg prednisone (or equivalent) or initiation of an immunomodulator or biologic occurred within 30 days of *C. difficile* testing. Therapies assessed included: azathioprine, methotrexate, anti-tumor necrosis factor (TNF) therapies, vedolizumab, ustekinumab and tofacitinib.

### Statistical analysis

The prevalence of toxin detection among positively screened patients was compared between individuals with IBD and those without IBD. Multivariable logistic regression was used to determine the association between a toxin positive assay and the available predictive variables of sex, race, inpatient location and IBD status. Among IBD patients, in order to test the hypothesis that stool toxin detection associates with the primary and secondary clinical outcomes of interest, we assessed the outcomes across the three categorical groups (screen positive/toxin positive, screen positive/toxin negative and screen negative). For continuous variables, means were compared using Kruskal–Wallis test for multiple groups and categorical variables were assessed using Pearson’s *χ*^2^ test. Tukey’s range test was used to compare differences across groups for continuous variables where indicated. A two-sided p-value < 0.05 was considered statistically significant. Statistical analysis was conducted using JMP **®** 13.1.0 (SAS Institute, Inc., Cary, NC).

## Results

### Prevalence of toxin detection in IBD vs the non-IBD population

Data was available from three of the four participating centers for all *C. difficile* positive screening tests including 1289 individuals among which 145 (11.2%) had IBD. Among patients with IBD, 22/145 (15.2%) were positive for toxin by EIA as compared to 413/1144 (36.1%) individuals from the non-IBD group (P < 0.0001). IBD patients were less likely to have a positive toxin testing as compared to the non-IBD group after controlling for inpatient status, sex and race (OR: 0.31, 95% CI: 0.19–0.51) in a multivariable model.

### Clinical outcomes in IBD patients based on toxin status

In total, 300 IBD patients from all four centers had a *C. difficile* assay performed during the study period with a median age of 49 years (IQR: 34–62) and IBD disease duration of 9.8 years (IQR: 4.5–18.3). Selected demographic characteristics and outcomes for IBD patients are listed in Table 1. In total, 25 (8.3%) patients had a screen positive assay/toxin positive assay, 136 (45.3%) had a screen positive/toxin negative assay and 139 (46.3%) had a negative screening assay. For the primary combined outcomes of interest and its individual components, no significant difference in outcomes was detected across the three groups (Table 2). Among individuals with a positive screening assay (n = 161), no significant difference in antimicrobial therapy was found between those with toxin detection and those without toxin detection. The primary initial treatment was vancomycin in 107 (68.6%) of the screen positive cases. Twenty-two (16.8%) screen positive/toxin negative results did not receive CDI specific treatment versus one (4%) of the screen positive/toxin positive cohort (p = 0.13). None of the 23 non-treated patients had a combined outcome of colectomy, ICU admission or death. Regarding the secondary outcomes, individuals with a screen positive/toxin negative test had significantly higher albumin and hemoglobin as compared to screen negative assays (p = 0.0008 and p = 0.0009, respectively). While not statistically significant, screen positive/toxin positive cases had a numerically higher white blood cell (WBC) count and C-reactive protein when compared to the screen positive/toxin negative and screen negative groups. When evaluated as a categorical variable, individuals with toxin detection had a numerically greater proportion of individuals with a WBC > 15 when compared to screen positive/toxin negative cases (6/18, 33% vs. 19/101, 18.8%, p = 0.164). Individuals with IBD and a negative screening test were more likely to have testing in the inpatient setting, however, no differences in admission or readmission rates were found across the three testing strategies. In total, 25% of individuals with IBD had therapy escalation after CDI testing without significant differences across testing strategies.

**Table 1:** Selected characteristics and outcomes of the 300 *C. difficile* assays performed in IBD patients. *: only assessed for screen positive cases

		Total n for subset
Age, years, median (IQR)	49 (34–62)	221
Male sex, n (%)	143 (47.7)	300
White race, n (%)	248 (82.7)	300
Inpatient location, n (%)	177 (59.2)	299
Screen positive/toxin positive, n (%)	25 (8.3)	300
Screen positive, toxin negative, n (%)	136 (45.3)	
Negative screen, n (%)	139 (46.3)	
Height, m, median (IQR)	1.7 (1.6–1.8)	142
Weight, kg, median (IQR)	75 (64.2–88.8)	172
BMI, kg/m^2^, median (IQR)	25.8 (22.2–30.4)	141
IBD duration, years, median (IQR)	9.8 (4.5–18.3)	275
Crohns disease, n (%)	160 (53.3)	300
Ulcerative colitis, n (%)	130 (43.3)	
IBD-unclassified, n (%)	3 (1)	
Pouch, n (%)	7 (2.3)	
History of prior CDI, n (%)	87 (29.1)	299
History of prior CDI in the past 8 weeks, n (%)	8 (4.6)	174
Active smoking, n (%)	29 (16.6)	175
IBD therapies		
Steroids, n (%)	106 (35.3)	300
IMM, n (%)	63 (21)	300
Anti-TNF, n (%)	81 (27)	300
Vedolizumab, n (%)	33 (11)	300
Ustekinumab, n (%)	16 (5.3)	300
Tofacitinib, n (%)	9 (3)	300
Proton pump inhibitor, n (%)	113 (37.7)	300
Antibiotic use in the past 4 weeks, n (%)	18 (10.3)	175
CDI Therapy*		
No treatment, n (%)	23 (14.7)	156
Metronidazole, n (%)	9 (5.8)	156
Vancomycin, n (%)	107 (68.6)	156
Metronidazole and vancomycin, n (%)	11 (7.1)	156
Fidaxomicin, n (%)	3 (1.9)	156
Fecal microbiota transplant, n (%)	3 (1.9)	156
Outcomes		
Death within 30 days, n (%)	4 (1.3)	300
Total colectomy or diverting ostomy, n (%)	11 (3.7)	300
ICU admission, n (%)	4 (1.3)	300
Primary combined outcome, n (%)	17 (5.7)	300
Any intraabdominal surgery, n (%)	23 (7.7)	300
Escalation in IBD therapy, n (%)	75 (25)	300
Laboratory values		
WBC, 10^3^/µL, median (IQR)	10.2 (7.4–14.7)	244
Hgb, g/dL, median (IQR)	10.6 (8.9–12.6)	245
Albumin, g/dL, median (IQR)	3.5 (2.9–4.1)	213
C-reactive protein, mg/L, median (IQR)	7.8 (3.1–37)	163

*BMI* body mass index, *CDI* *C. difficile* infection, *Hgb* hemoglobin, *IBD* inflammatory bowel disease, *IMM* immunomodulator, *IQR* interquartile range, *kg* kilograms, *m* meters, *TNF* tumor necrosis factor, *WBC* white blood cell

**Table 2:** Clinical outcomes based on *C. difficile* assay result among patients with inflammatory bowel disease

Outcomes	Screen positive/toxin positive, n = 25	Screen positive/toxin negative, n = 136	Negative screen, n = 139	P-value
CDI Therapy				
No treatment, n (%)	1 (4)	22 (16.8)	-	0.128
Metronidazole, n (%)	2 (8)	7 (5.1)	-	0.665
Vancomycin, n (%)	20 (80)	87 (64)	-	1
Metronidazole and vancomycin, n (%)	1 (4)	10 (7.4)	-	0.688
Fidaxomicin, n (%)	1 (4)	2 (1.5)	-	0.41
Fecal microbiota transplant, n (%)	0 (0)	3 (2.2)	-	1
Primary and secondary outcomes				
Death within 30 days, n (%)	0 (0)	2 (2.8)	2 (1.4)	0.715
Total colectomy or diverting ostomy, n (%)	0 (0)	3 (2.2)	8 (5.8)	0.175
ICU admission, n (%)	1 (4)	2 (1.5)	1 (0.7)	0.413
Primary combined outcome, n (%)	1 (4)	6 (4.4)	10 (7.2)	0.566
Escalation in IBD therapy, n (%)	7 (28)	29 (21.3)	39 (28.1)	0.408
Inpatient setting, n (%)	**12 (48)**	**62 (45.9)**	**103 (74.1)**	** < 0.0001**
Inpatient LOS, d, mean (SD)	11.6 (22.2)	8 (9)	11.8 (14.1)	0.395
Admission to the hospital among outpatients, n (%)	1 (8.3)	7 (10.1)	3 (8.3)	0.947
Readmission to the hospital among inpatients, n (%)	2 (16.7)	13 (19.4)	15 (14.7)	0.724
Laboratory values				
WBC, 10^3^/µL, mean (SD)	13.2 (10.7)	11 (5.8)	11.9 (6.2)	0.613
Hgb, g/dL, mean (SD)	**11.6 (2.7)**	**11.4 (2.6)**	**10.2 (2)**	**0.001**
Albumin, g/dL, mean (SD)	**3.5 (0.9)**	**3.6 (0.8)**	**3.2 (0.9)**	** < 0.001**
C-reactive protein, mg/L, mean (SD)	82.5 (113.8)	30.3 (53.3)	35.1 (55.5)	0.593

*CDI* *C. difficile* infection, *Hgb* hemoglobin, *IBD* inflammatory bowel disease, *ICU* intensive care unit, *LOS* length of stay, *WBC* white blood cell, **Bold:** P < 0.05

## Discussion

The 2017 Infectious Diseases Society of America (IDSA) and Society for Healthcare Epidemiology of America (SHEA) clinical guideline for the management of *C. difficile* recommends a two-step testing strategy for *C. difficile* when there are no preagreed institutional criteria for patient stool submission [5]. Namely, a screening glutamate dehydrogenase (GDH) assay or NAAT assay followed by an expressed toxin test is recommended over a toxin assay alone [4] due to the reduced sensitivity of the expressed toxin test when used as a standalone assay [6]. In order to determine the impact of *C. difficile* testing results on patients with IBD, we assessed the prevalence of toxin expression in assays performed on patients with IBD and secondly compared clinically relevant outcomes between IBD patients with positive and negative toxin testing.

When compared to the non-IBD population, individuals with IBD are less likely to have toxin detected by EIA when the screening GDH or NAAT test is positive. While, toxin EIA assays may be less sensitive for toxin detection in the stool of patients with IBD [7], a recent study by Sokol et al., found that none of the screen positive/toxin negative assays from patients with IBD had positive toxin detection by cytotoxicity assay, which suggests the true absence of clinically active toxin [8]. When considering centers that perform a NAAT screening assay as their standalone test for the diagnosis of CDI, this also represents an overtreatment of *C. difficile* in patients with IBD as the majority of patients with IBD with a positive screening assay do not have toxin expression detected.

Among patients with IBD, when comparing patients with screen positive/toxin positive tests to those with screen positive/toxin negative tests and negative screening tests, no significant differences were found with respect to the clinical outcomes of interest or hospitalization/readmission. In fact, individuals with negative screening tests were more likely to have lower hemoglobin and albumin levels, although these differences corrected after controlling for age and inpatient status (see Additional file 2). These results are in line with prior studies in IBD in which detection of CDI by NAAT (screening strategy) or enzyme-linked immunosorbent assay (ELISA) for toxin did not demonstrate differences in the clinical outcomes of ICU admission, hospital LOS, surgery or readmission [9]. A recent study by Gupta et al. found that toxin positivity did not associate with IBD complications, or the laboratory values of WBC, albumin or C-reactive protein [10]. However, toxin positivity did associate with response to CDI therapy, which was unable to be assessed in our retrospective collection [10]. Future studies will be required to assess the role of more sensitive toxin assays in IBD and the potential impact of CDI specific therapies on clinical outcomes.

When focusing on individuals with a positive screening assay and a negative toxin assay (discordant result), the IDSA clinical guideline for the management of *C. difficile* offers supporting evidence without strict guidance on the management of such individuals [5]. Similarly, the American College of Gastroenterology clinical guideline on the diagnosis and treatment of CDI recommends clinical evaluation and consideration of colonization as opposed to infection in such cases [11]. In the largest study to date from the United Kingdom, Planche et al. performed cytotoxigenic culture and cytotoxin assays to assess for the presence of *C. difficile* and expressed toxin, respectively. Among 6522 tested isolates from inpatient samples, mortality was highest among individuals with expressed toxin as compared to cytotoxin negative assays and individuals with negative cytotoxigenic culture [12]. In a single center study from the United States, Polage et al., demonstrated that screen positive/toxin negative patients had a shorter duration of diarrhea and no CDI-related complications when compared to screen positive/toxin positive assays [3]. Similar to our results from the non-IBD cohort, only 44.7% of individuals with screen positive assays had toxin detection. Different from our study was the fact that minimal empiric treatment for CDI was present in the screen positive/toxin negative cases. In our study, in patients with IBD, the majority of screen positive/toxin negative (n = 114/136) cases received treatment for CDI. Importantly, in the small subgroup of patients with IBD and screen positive/toxin negative assays that did not receive CDI specific therapy, no patients experienced a complication of their disease. Therefore, future prospective studies should focus on limiting treatment of screen positive/toxin negative cases in IBD in order to reduce the unnecessary burdens and potential harms of excessive antibiotic exposure.

The primary limitation of our study is the retrospective collection of data from multiple sites and therefore inability to control for testing decisions or control management decisions related to CDI therapy in patients with IBD. With respect to testing decisions, ascertainment bias can result if patients with IBD are increasingly tested for CDI leading to a lower probability of toxin detection due to a higher rate of colonization of *C. difficile* in IBD [1]. We attempted to control for some testing strategy differences in our initial analysis of patients with IBD compared to the non-IBD population by performing a multivariate analysis that included inpatient status in the analysis. With respect to the clinical outcomes in individuals with IBD, given the multicenter nature of the study, selection bias can result related to differences in testing criteria between centers which would impact the primary outcome. This can be demonstrated in our data in which individuals with a negative screening assay with IBD had lower hemoglobin values and albumin values at the time of diagnosis which corrected after adjustment for baseline differences in testing location and patient age. Lastly, the retrospective nature of the study limited our ability to formally collect symptoms before testing for CDI and to fully understand the risk factors for CDI such as prior antibiotic use. Prior studies have shown up to 14% of individuals with asymptomatic carriage can have toxin detected in their stool by EIA highlighting potential false positive cases of CDI even with toxin detection and the need for an assessment of clinical symptoms prior to testing and during treatment [13].

In conclusion, we demonstrate that there is reduced detection of expressed *C. difficile* toxin from patients with IBD patients when compared to the non-IBD population. This represents an overtreatment of CDI in IBD when NAAT-based testing strategies are used alone without a multistep testing algorithm. While we were unable to demonstrate significant differences in clinical outcomes based on toxin detection in IBD, this is consistent with prior studies and is unfortunately limited by the rare occurrence of the primary outcome among patients with toxin expression. Future prospectively collected studies should focus on the response to treatment of CDI when diagnosed using multistep algorithms including toxin expression and the clinical outcomes of individuals with screen positive/toxin negative assays not receiving treatment in IBD.

## Supplementary Information

**Additional file 1: Table S1.** Two-step assays utilized during the retrospective study from participating centers. **Table S2.** Breakdown of CDI testing from IBD patients from participating centers.

**Additional file 2: Table S1.** ANOVA table for full model including toxin status, inpatient status and age for hemoglobin. Accompanying graph shows hemoglobin values across toxin status for both inpatients and outpatients. **Table S2.** ANOVA table for full model including toxin status, inpatient status and age for albumin. Accompanying graph shows serum albumin values across toxin status for both inpatients and outpatients.

## Data Availability

Because our data contain personal identifiers, requests must be made to the individual participating Institutional Review Boards.

## Abbreviation

VCE: Video capsule endoscopy
