# Supplementary material for: Clostridioides difficile toxin is infrequently detected in inflammatory bowel disease and does not associate with clinical outcomes
Source: Gut Pathog. 2022 Aug 30;14:36. doi: 10.1186/s13099-022-00511-2 (PMC9426007; doi:10.1186/s13099-022-00511-2)
Supplement: Supplementary file 1 — Additional file 1: Table S1. Two-step assays utilized during the retrospective study from participating centers. Table S2. Breakdown of CDI testing from IBD patients from participating centers. [file 13099_2022_511_MOESM1_ESM.docx]

**Additional file 1:**

**Methods:**

Initial sample testing was performed at the discretion of the clinical care teams from four US academic medical centers: Loma Linda University (LL), University of Michigan (UM), University of Louisville (UofL) and Vanderbilt University (VU).

The individual participating institutions had slight variations on the testing strategy but consisted of an initial screening assay for either glutamate dehydrogenase (GDH) (UM) or detection of target toxin genes by NAAT (LL, UofL, VU) as noted in the accompanying **Additional file 1: Table S1**. A second-step assay was performed for the confirmation of toxin detection in the stools of positively screened patients by enzyme immunoassay (EIA) allowing for the separation of patients into the categories: screen positive/toxin positive, screen positive/toxin negative or negative screening assay.

Screen positive cases were separately available for the general population from LL, UM and VU. Screen negative cases from IBD patients were separately available from UM (1:10 randomly selected negative cases) and UofL (**Additional file 1: Table S2**).

**Table S1:** Two-step assays utilized during the retrospective study from participating centers.

|  | **Screening** | **Screening method (**glutamate dehydrogenase (GDH) or NAAT) | **Toxin EIA** | **Additional assays** |
| --- | --- | --- | --- | --- |
| **UM** | C. DIFF QUIK CHEK COMPLETE® (Techlab, Inc., Blacksburg, VA) | GDH | C. DIFF QUIK CHEK COMPLETE® (Techlab, Inc., Blacksburg, VA) | BD GeneOhm™ Cdiff Assay; Franklin Lakes, NJ |
| **LL** | Xpert® *C. difficile* (Cepheid, Sunnyvale, CA) | NAAT | Immunocard® Toxins A&B (Meridian Bioscience, Cincinnati, OH) |  |
| **UofL** | Xpert® *C. difficile* (Cepheid, Sunnyvale, CA) | NAAT | C. DIFF QUIK CHEK COMPLETE® (Techlab, Inc., Blacksburg, VA) |  |
| **VU** | Simplexa^TM^ *C. difficile* Direct Assay (Diasorin Molecular LLC, Cypress CA) | NAAT | C. DIFF QUIK CHEK COMPLETE® (Techlab, Inc., Blacksburg, VA) |  |

**Table S2:** Breakdown of CDI testing from IBD patients from participating centers.

|  | **Screen positive/toxin positive (n)** | **Screen positive/toxin negative (n)** | **Negative screen (n)** |
| --- | --- | --- | --- |
| **UM** | 7 | 49 | 69 |
| **LL** | 0 | 10 |  |
| **UofL** | 3 | 13 | 70 |
| **VU** | 15 | 64 |  |
| **Total** | 25 | 136 | 139 |
