# Supplementary material for: Clostridioides difficile toxin is infrequently detected in inflammatory bowel disease and does not associate with clinical outcomes
Source: Gut Pathog. 2022 Aug 30;14:36. doi: 10.1186/s13099-022-00511-2 (PMC9426007; doi:10.1186/s13099-022-00511-2)

**Additional file 2:**

**Table S1: ANOVA table for full model including toxin status, inpatient status and age for hemoglobin. Accompanying graph shows hemoglobin values across toxin status for both inpatients and outpatients.**

| Source of variation | DF | Sum of Squares | F Ratio | P-value |
| --- | --- | --- | --- | --- |
| Toxin status | 2 | 1.58 | 0.21 | 0.812 |
| Inpatient status | 1 | 163.44 | 43 | <0.001 |
| Age | 1 | 31.93 | 8.4 | 0.004 |


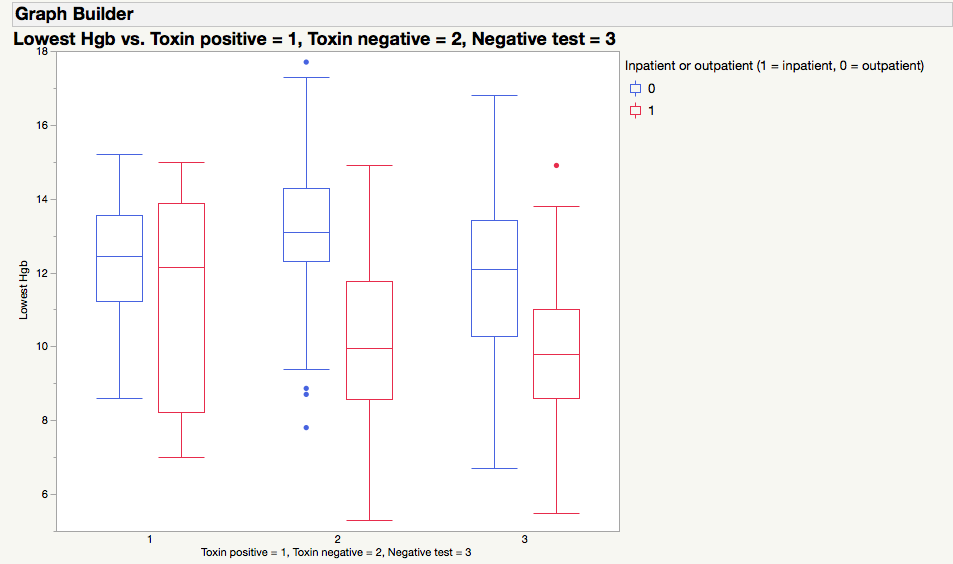


**Table S2: ANOVA table for full model including toxin status, inpatient status and age for albumin. Accompanying graph shows serum albumin values across toxin status for both inpatients and outpatients.**

| Source of variation | DF | Sum of Squares | F Ratio | P-value |
| --- | --- | --- | --- | --- |
| Toxin status | 2 | 0.91 | 0.81 | 0.447 |
| Inpatient status | 1 | 15.06 | 26.64 | <0.001 |
| Age | 1 | 9.83 | 17.39 | <0.001 |


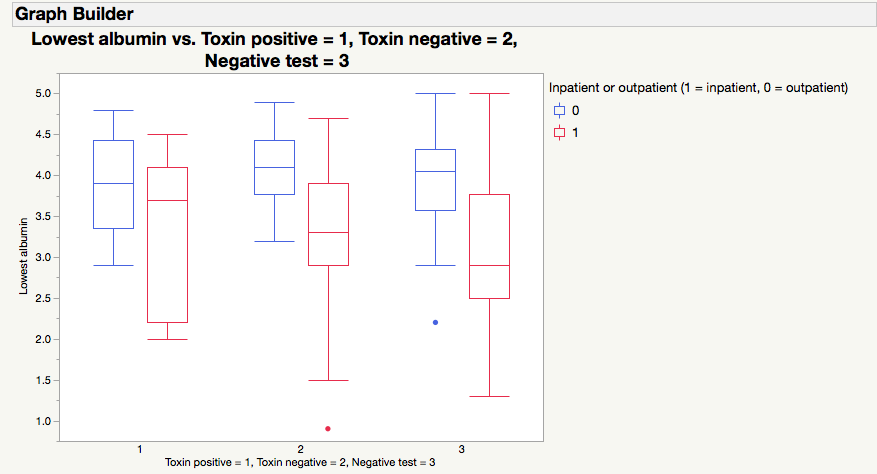

Supplement: Supplementary file 2 — Additional file 2: Table S1. ANOVA table for full model including toxin status, inpatient status and age for hemoglobin. Accompanying graph shows hemoglobin values across toxin status for both inpatients and outpatients. Table S2. ANOVA table for full model including toxin status, inpatient status and age for albumin. Accompanying graph shows serum albumin values across toxin status for both inpatients and outpatients. [file 13099_2022_511_MOESM2_ESM.docx]
